# Anorectal Melanoma: A Rare Cause of Large Bowel Obstruction

**DOI:** 10.7759/cureus.56128

**Published:** 2024-03-13

**Authors:** Anuradha S Dnyanmote, Suhasini Jadhav, Kinjal Vasava, Saikumar Immadi

**Affiliations:** 1 Surgery, Dr. D. Y. Patil Medical College, Hospital and Research Centre, Pune, IND; 2 General Surgery, Dr. D. Y. Patil Medical College, Hospital and Research Centre, Pune, IND

**Keywords:** malignant melanoma, abdominal perineal resection, large bowel obstruction, anorectal melanoma, melanoma

## Abstract

Anorectal melanoma is a rare and aggressive malignancy with a challenging diagnosis and management. We present the case of a 69-year-old male with a history of chronic constipation and recent weight loss, who presented with symptoms suggestive of anorectal pathology. Despite initial diagnostic challenges, including an unsuccessful colonoscopy due to inadequate bowel preparation, the subsequent radiographic evaluation revealed a large bowel obstruction secondary to a protruding anorectal mass. Abdominal X-ray demonstrated significant colonic dilation while contrast-enhanced CT scan revealed a large hypodense mass protruding from the anal canal. Surgical intervention led to the excision of the mass, and histopathological examination confirmed malignant melanoma. Immunohistochemistry markers, including HMB 45, Melan A, and S100, supported the diagnosis. This case underscores the importance of considering anorectal melanoma in patients presenting with atypical anorectal symptoms, despite its rarity. Early recognition and intervention, supported by appropriate imaging modalities, are critical for optimizing patient outcomes in such cases.

## Introduction

Anorectal mucosal melanoma (AMM) is an extremely rare and aggressive neoplasm, comprising only 0.4% to 1.6% of all melanomas and 0.5% of all anorectal cancers [1]. It predominantly affects older individuals, usually in their sixth to eighth decades of life, and is more commonly observed in white females [2]. Malignant melanoma commonly occurs in the anorectum as the third most frequent location, following the skin and retina. The presentation of AMM is often nonspecific, with symptoms such as pain, bleeding, anal mass, tenesmus, or constipation. Consequently, the diagnosis is frequently delayed, leading to a poor prognosis [3]. AMM is frequently misdiagnosed as hemorrhoids, polyps, or colorectal cancer due to its rarity and atypical symptoms [3]. Surgical resection is the mainstay of treatment for AMM, although the optimal procedure remains a subject of debate. Both wide local excision (WLE) and abdominal perineal resection (APR) are viable options [4]. The necessity of regional lymphadenectomy is a topic of debate; however, it is advised to conduct a sentinel lymph node biopsy for all localized melanomas. There is limited research conducted on this rare condition, resulting in a scarcity of available data for physicians and researchers. Timely and accurate diagnosis, followed by multidisciplinary management, plays a crucial role in improving the quality of life and prognosis of patients affected by AMM [5].

## Case presentation

A 69-year-old male presented to the hospital with complaints of incomplete evacuation of stool and swelling protruding from the perianal region, which had been occurring intermittently for the past four months. The patient had a history of passing hard stools and straining but recently developed loose stools after taking Ayurvedic medication for constipation. Additionally, he reported a history of incontinence for stool and a significant weight loss of 12 kilograms over three months. The patient had hypertension and no other comorbidities. There was no history of carcinoma in the family, but the patient was a chronic tobacco chewer for the last 25 years.

**Figure 1:**
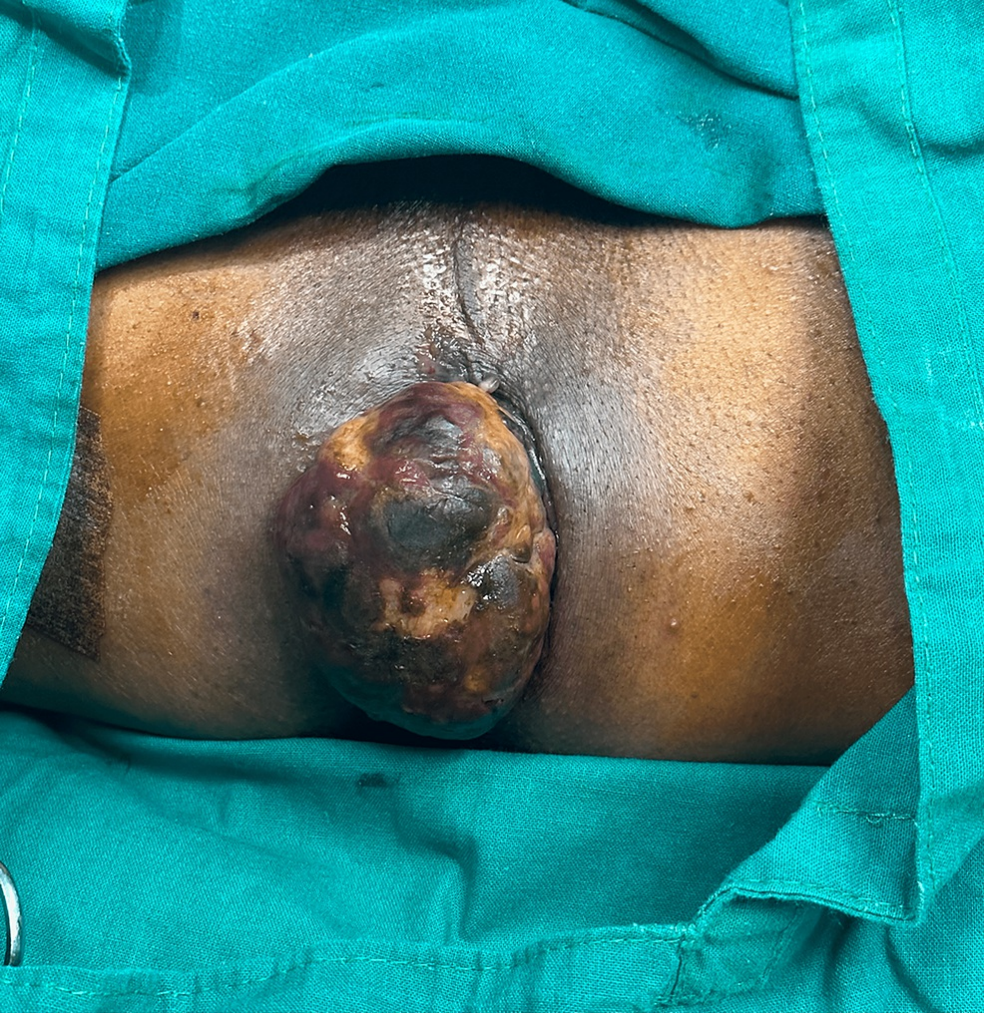
Protrusion of the anorectal mass The protruding mass from the anal canal shows black pigmentation.

Upon examination, external skin tags were observed at the 6 and 12 o'clock positions, along with a palpable fissure at the same location. The sphincter tone was lax, and there was no active bleeding. Proctoscopy revealed a grade 2 prolapsed internal hemorrhoid at the 10 o'clock position. Routine blood investigations were within normal limits. Further investigation was done by a diagnostic colonoscopy after giving laxatives for bowel preparation. However, despite the preparation, the patient continued to complain of an inability to evacuate stool. A colonoscopy could not be performed due to inadequate bowel preparation. While straining the patient suddenly experienced bright red bleeding per rectum and a mass protruding from the anal verge. Upon examination, a firm, globular, nontender, irreducible pedunculated mass measuring approximately 6 x 4 cm was found extending from the 2 to 5 o'clock positions (Figure 1).

**Figure 2:**
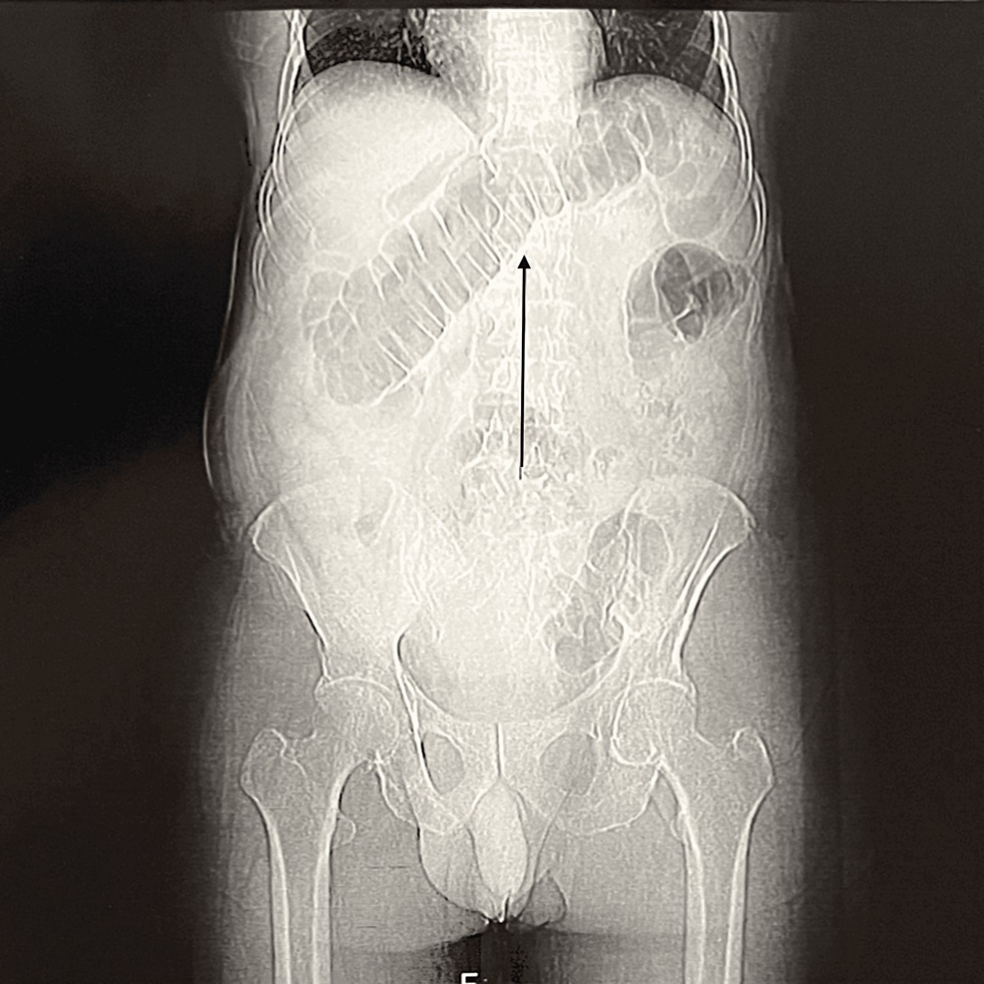
Coronal section showing a large bowel obstruction The black arrow indicates a fully loaded transverse colon

**Figure 3:**
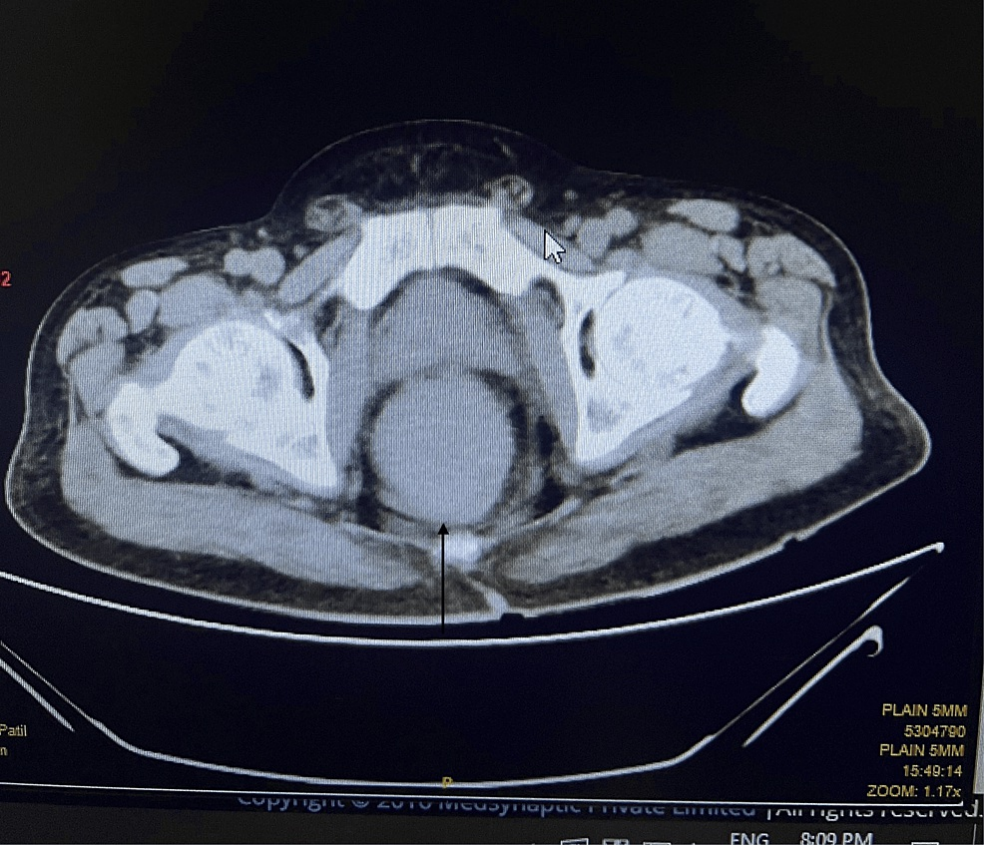
The transverse section shows a mass at the anorectal junction The black arrow indicates a mass in the anorectal junction.

Contrast-enhanced CT scan of the abdomen and pelvis revealed a large bowel obstruction with significant dilatation of the ascending, transverse, descending, sigmoid colon, and rectum. The scan also identified a large, hypodense, oval-shaped eccentric mass along the left lateral wall of the anal canal, which protruded outside the anal verge. The mass measured 4.7 x 6.1 x 7.8 cm, with thickening of the mesorectal fascia on the left side. No perirectal lymph nodes were detected in the scan (Figures 2, 3).

**Figure 4:**
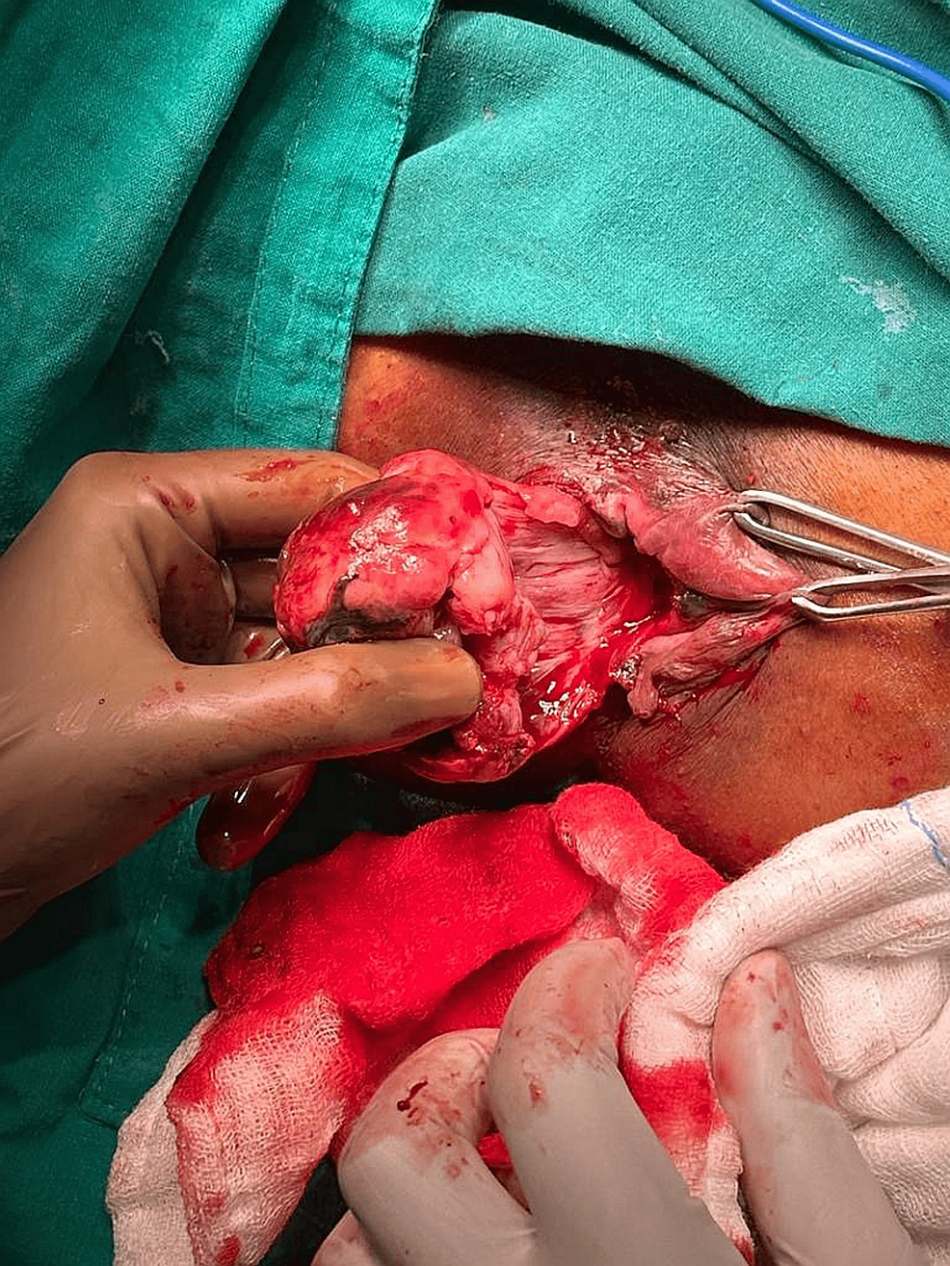
Pedenculated rectal mass below the dentate line The image indicates a mass raising from the left lateral wall of the anal canal.

**Figure 5:**
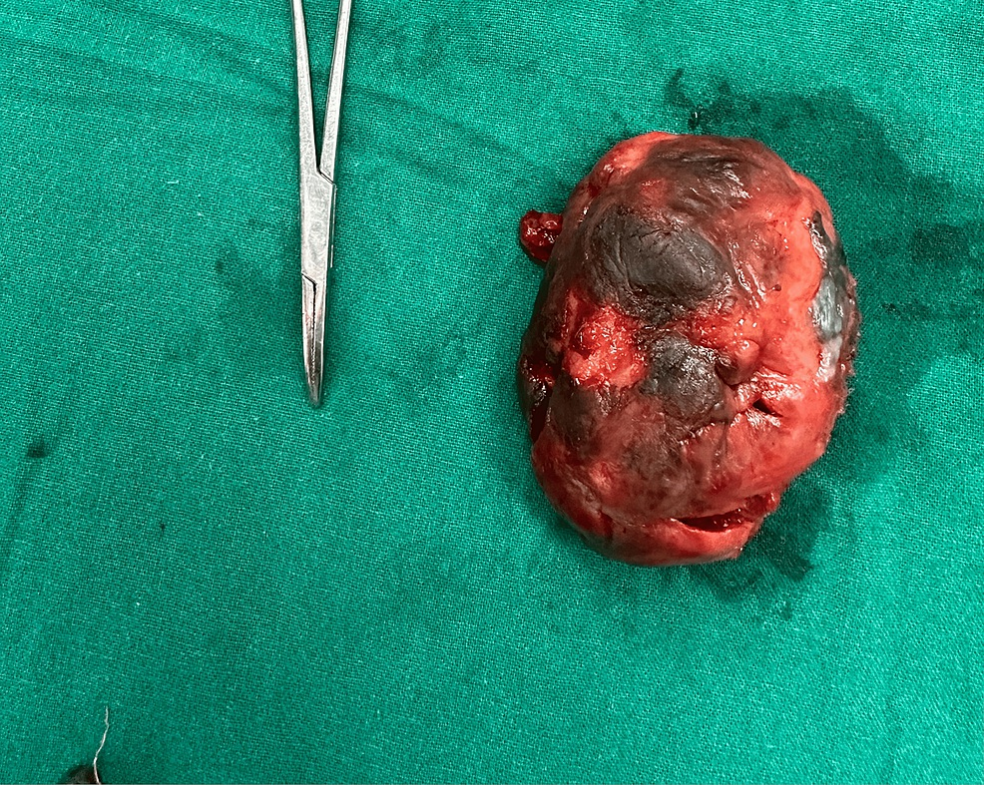
Excised specimen of the anorectal mass The medial surface of the tumor shows black pigmentation.

Due to dynamic obstruction caused by the protruding mass, the patient was taken up for emergency surgery, and excision of the rectal mass with mucosal flap closure was performed. A specimen of excision biopsy was sent for a histopathology examination (Figures 4, 5).

**Figure 6:**
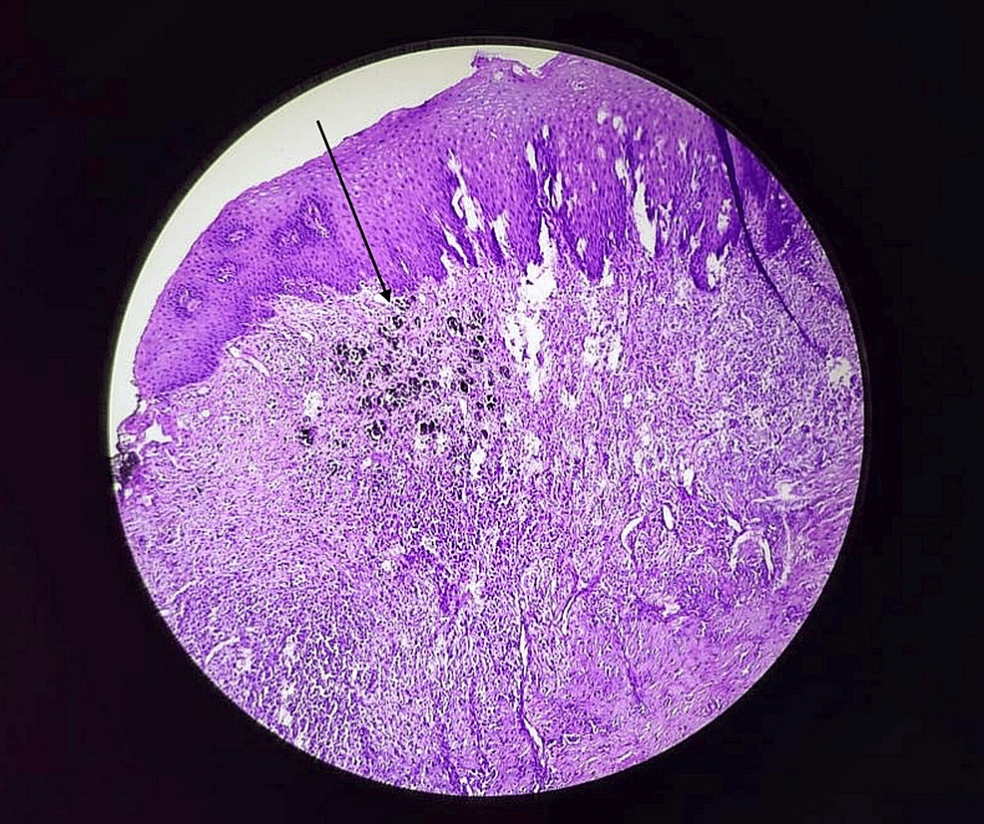
Histopathological examination The black arrow indicates brown pigment below the stratified squamous epithelium. Neoplastic cells are arranged in the sheets and nests pattern.

**Figure 7:**
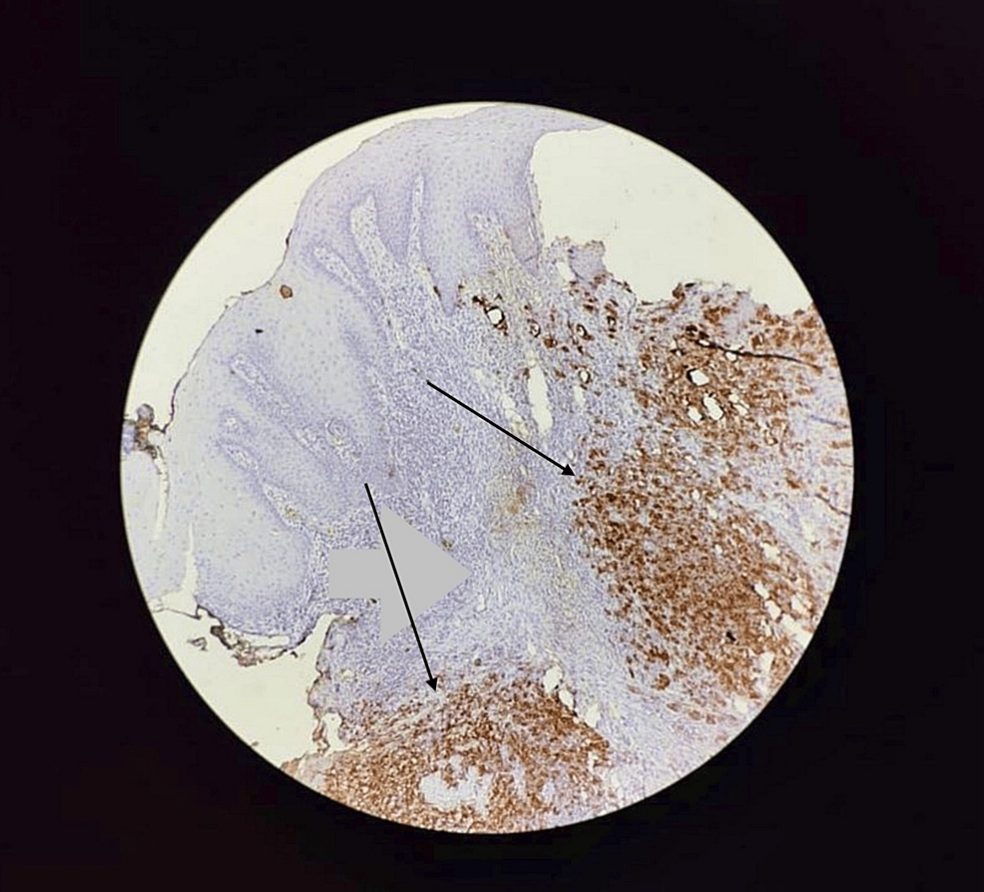
Melan A positive Melan A is a cytoplasmic stain that detects a melanocyte-specific cytoplasmic protein. This image indicates Melan A is positive for this tumor.

Histopathological examination of multiple sections from the polypoidal mass revealed neoplastic cells arranged in sheets and nests. These cells appeared large, pleomorphic, hyperchromatic, and had prominent nucleoli with eosinophilic cytoplasm. A brownish pigment was noted in cells located below the lining stratified squamous epithelium. The neoplastic cells infiltrated the muscle layer and abutted the resection margin. Additionally, the surface of the polypoidal lesion showed areas of hemorrhage and mixed inflammatory cell infiltration. The histopathology examination of the excised specimen confirmed the diagnosis of malignant melanoma (Figures 6, 7).

After confirmation of this rare malignancy, the patient was evaluated for the metastatic state of the disease. Positron emission tomography-computed tomography (PET-CT) revealed no hypermetabolic activity seen anywhere in the body except for the anorectal junction. The tumor showed positive immunohistochemistry (IHC) markers for HMB 45, Melan A, and focal positivity for S100. 

After confirmation of the diagnosis of anorectal melanoma without any metastasis, abdominal perineal resection was done with permanent colostomy. The patient was started on intravenous chemotherapy with dacarbazine after four weeks of surgery.

The combination of clinical presentation, histopathology findings, and positive IHC markers helps establish a definitive diagnosis of anorectal melanoma.

## Discussion

Melanocytes, originating from the neural crest or mucocutaneous junctions, play a crucial role in melanin synthesis and exhibit antioxidant activity in mucosal areas [6-8]. The anal canal is derived from the handout proximally by the upper limit of the anal transition zone. The anal canal has three zones: colorectal zone, transitional zone, and squamous zone. Melanocytes can be found in the annual squamous zone, sometimes in the anal transitional zone, but never in the colorectal zone. Melanocytes migrate via the umbilical-mesenteric canal and later distribute into specialized cells that undergo malignant transformation [9]. The malignant transformation of anorectal areas, such as in anorectal melanoma (AM), may be associated with oxidative stress or immunosuppression in these regions [6]. Alternatively, theories propose that AM, along with gastrointestinal melanoma, may arise from Schwannian neuroblastic cells of the autonomic intestinal innervation system or cells of the amine-precursor uptake and decarboxylation (APUD) system of the gut [7]. The APUD system comprises cells derived from the neuroectoderm that migrate during embryogenesis to form various structures, including the skin [7,10]. These cells are involved in the uptake and production of biologically active amines [7]. While AM often occurs in patients aged >50 years, the exact etiology and mechanisms underlying its development require further exploration [6,7].

Early diagnosis of anorectal melanoma (AM) is challenging due to its hidden location and lack of specific symptoms, leading to delayed diagnosis and poor prognosis [11]. AM is often characterized by large tumoral masses (>3-4 cm) and presents with changes in bowel habits, bowel obstruction, rectal bleeding, anal pain, and rectal tenesmus [12,13]. Clinical examination reveals polypoid, ulcerated, non-pigmented lesions with an irregular surface, sometimes showing black or brown spots [14]. The amelanotic presentation of AM can mimic other gastrointestinal diseases, resulting in misdiagnosis [14]. Accurate instrumental investigations, such as rectal examination, endoscopic ultrasonography, proctosigmoidoscopy, and histological evaluation with immunohistochemistry (HMB-45, S-100, Melan-A, Vimentin), are crucial for diagnosis [14]. Supplementing colonoscopy with endoscopic ultrasound and MRI aids in assessing tumor infiltration and staging [6,15]. The lack of standardized diagnostic protocols highlights the need for improved diagnostic methods to enhance sensitivity and specificity in detecting AM [16]. Metastases occur via lymphatic and hematogenous routes, and it has been reported that 38% of patients already have metastatic disease at the time of diagnosis. Lymphatic spread to mesenteric nodes is more common than to inguinal nodes while lungs, liver, and bones are the most frequent sites of distant metastases [17].

Diagnosing mucosal melanoma can be challenging due to delayed presentation, ulceration, and the absence of pigmentation. Junctional changes may not be present in ulcerated lesions, making it difficult to differentiate between primary and metastatic disease [14,18]. The histologic types of AM include epithelioid, spindle-cell, lymphoma-like, and pleomorphic AM [18]. Immunohistochemical analysis, particularly with S100, Melan-A, HMB-45, and tyrosinase, is crucial for accurate diagnosis [18]. S100 is highly sensitive but lacks specificity while Melan-A, HMB-45, and tyrosinase show higher specificity for melanocytic lesions [18]. Pan-cytokeratin is usually negative, but rare cases of melanoma expressing epithelial markers can occur [18]. Caution is needed to avoid misdiagnosis with poorly differentiated rectal carcinoma [18]. SOX10 is a nuclear transcription factor that plays an important role in melanocytic cell differentiation. It has been shown to be a sensitive marker of melanoma. The sensitivity and specificity for SOX10 in the diagnosis of melanoma are 1.0 and 0.93, respectively [19]. Additionally, numerous studies have revealed the cause of this cancer as related to various somatic driver mutations, including alterations in KIT-a proto-oncogene encoding for a transmembrane receptor tyrosine kinase. Although accounting for only 3% of all melanomas, mutations in c-KIT are mostly derived from acral, mucosal, and chronically sun-damaged melanomas [20]. c-Kit is positive in about 75% of AMs and can aid in diagnosis [18].

Surgery is the primary treatment for mucosal melanoma (AM), but it does not significantly improve overall survival. Abdominoperineal resection (APR) does not show survival benefits compared to local excision, although it may improve disease-free survival for loco-regional control [21,22]. The extent of surgical excision does not influence long-term outcomes, and local excisions can be recommended for selected cases to improve quality of life [22]. Although regional lymphadenectomy's role is debated, sentinel lymph node biopsy is recommended for all localized melanomas. This helps in proper staging [23]. Adjuvant radiotherapy can be used for local disease control, but lymph node irradiation is controversial due to the risk of lymphedema [22]. Systemic therapies for AM lack consensus. Traditional chemotherapy, including dacarbazine and temozolomide, shows limited efficacy with partial responses and short response durations [23,24]. Combination chemotherapy regimens have not demonstrated significant survival benefits over single-agent therapy [25]. The MSLT-II study established the current standard of care for patients with stage III melanoma in favor of sentinel lymph node dissection, followed by sonographic observation for five years. In addition to surgery, we also offer one year of adjuvant therapy with either a checkpoint inhibitor or BRAF/MEK-inhibitors (only where the tumor exhibits the BRAF-V600E/K mutation) to patients with high-risk stage III and completely resected stage IV melanoma [26]. Targeted therapies, specifically tyrosine kinase inhibitors (TKIs) for c-Kit mutations, have shown effectiveness, including imatinib and sorafenib [27,28]. Immune checkpoint inhibitors like ipilimumab, nivolumab, and pembrolizumab have shown promise in AM treatment [29,30]. Combination therapies involving chemotherapy, targeted therapy, and immune checkpoint inhibitors are important options for AM patients [9,30].

## Conclusions

Anorectal melanoma (AM) is a rare and aggressive neoplasm with nonspecific symptoms, often leading to delayed diagnosis and poor prognosis. An accurate diagnosis of AM requires a combination of clinical examination, histopathological examination, and immunohistochemistry analysis using markers such as HMB 45, Melan A, and S100. Surgical resection is the mainstay of treatment, with the optimal procedure still under debate. Adjuvant radiotherapy and systemic therapies, including chemotherapy, targeted therapy, and immune checkpoint inhibitors, may be considered in advanced cases. Prompt diagnosis and multidisciplinary management are crucial in improving outcomes for patients with anorectal melanoma.

Here, we presented a rare case of anorectal melanoma that was treated with surgical excision followed by chemotherapy. This case underscores the importance of considering anorectal melanoma in patients presenting with atypical anorectal symptoms, despite its rarity. Early recognition and intervention, supported by appropriate imaging modalities, are critical for optimizing patient outcomes in such cases.
